# Kai-Xin-San Inhibits Tau Pathology and Neuronal Apoptosis in Aged SAMP8 Mice

**DOI:** 10.1007/s12035-021-02626-0

**Published:** 2022-03-18

**Authors:** Ya-Nan Jiao, Jing-Sheng Zhang, Wen-Jun Qiao, Shu-Yu Tian, Yi-Bin Wang, Chun-Yan Wang, Yan-Hui Zhang, Qi Zhang, Wen Li, Dong-Yu Min, Zhan-You Wang

**Affiliations:** 1 Health Sciences Institute, China Medical University, Shenyang, China; 2 Affiliated Hospital of Liaoning University of Traditional Chinese Medicine, Shenyang, China; 3 School of Fundamental Sciences, China Medical University, Shenyang, China

**Keywords:** Kai-Xin-San, Network pharmacology, Tau hyperphosphorylation, Inflammation, Apoptosis

## Abstract

Alzheimer’s disease (AD) is an age-related neurological disorder. Currently, there is no effective cure for AD due to its complexity in pathogenesis. In light of the complex pathogenesis of AD, the traditional Chinese medicine (TCM) formula Kai-Xin-San (KXS), which was used for amnesia treatment, has been proved to improve cognitive function in AD animal models. However, the active ingredients and the mechanism of KXS have not yet been clearly elucidated. In this study, network pharmacology analysis predicts that KXS yields 168 candidate compounds acting on 863 potential targets, 30 of which are associated with AD. Enrichment analysis revealed that the therapeutic mechanisms of KXS for AD are associated with the inhibition of Tau protein hyperphosphorylation, inflammation, and apoptosis. Therefore, we chose 7-month-old senescence-accelerated mouse prone 8 (SAMP8) mice as AD mouse model, which harbors the behavioral and pathological hallmarks of AD. Subsequently, the potential underlying action mechanisms of KXS on AD predicted by the network pharmacology analyses were experimentally validated in SAMP8 mice after intragastric administration of KXS for 3 months. We observed that KXS upregulated AKT phosphorylation, suppressed GSK3β and CDK5 activation, and inhibited the TLR4/MyD88/NF-κB signaling pathway to attenuate Tau hyperphosphorylation and neuroinflammation, thus suppressing neuronal apoptosis and improving the cognitive impairment of aged SAMP8 mice. Taken together, our findings reveal a multi-component and multi-target therapeutic mechanism of KXS for attenuating the progression of AD, contributing to the future development of TCM modernization, including KXS, and broader clinical application.

**Graphical abstract:**

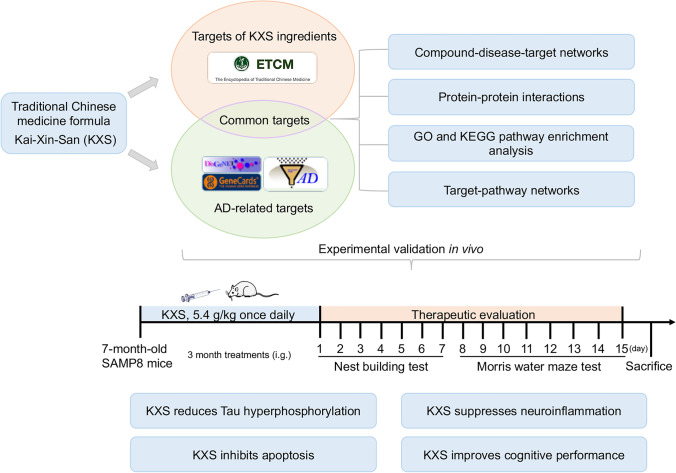

**Supplementary Information:**

The online version contains supplementary material available at 10.1007/s12035-021-02626-0.

## Introduction

Alzheimer’s disease (AD) is an age-related neurodegenerative disease, with symptoms of progressive cognitive decline and memory impairment. In 2019, fifty million people worldwide have been affected by dementia according to the World Alzheimer Report, and the number is predicted to reach 152 million by 2050, of which AD accounts for 50–75% [1]. Currently, there is no effective cure for AD. There remain urgent needs for therapeutic methods to slow the progress of the diseases.

AD is pathologically characterized by intercellular β-amyloid (Aβ) plaques and intracellular neurofibrillary tangles (NFTs) [2]. NFTs are composed of straight and paired-helical filaments (PHFs), both of which are composed predominantly of insoluble polymers of abnormally hyperphosphorylated microtubule-associated protein Tau [3]. In AD brains, Tau is aberrantly hyperphosphorylated, carrying a threefold to fourfold of phosphates [4]. The abnormal Tau phosphorylation seems to be related to the alteration of several kinases, including glycogen synthase kinase 3 beta (GSK3β), cyclin-dependent kinase 5 (CDK5), cAMP-dependent protein kinase (PKA), and microtubule-affinity-regulating kinase (MARK) [5]. GSK3β and CDK5 are the kinases primarily responsible for phosphorylation of Tau. GSK3β-phosphorylated sites of Tau include the Thr181, Thr231, Ser396, Ser404, and Ser202 [6]. GSK3β (Ser9) is a well-known Akt phosphorylation site, and the Ser9 phosphorylation reduces the kinase activation of GSK3β, whereas phosphorylation of GSK3β on Tyr216 leads to activation [7, 8]. The binding of p35 to Cdk5, and phosphorylation on Tyr15, can activate the Cdk5 kinase which in turn phosphorylates Tau [9, 10]. Therefore, GSK3β and CDK5 inactivation prevent Tau phosphorylation and are crucial to the development of AD. Besides Aβ plaques and NFTs in AD, neuroinflammation has been increasingly implicated as a major contributing factor to AD [11, 12], meanwhile inducing more Tau phosphorylation [13]. The use of existing drugs to target neuroinflammation has been proved effective in AD mouse models and patients; therefore, anti-inflammatory may represent a potential therapeutic strategy for AD [14–18].

In recent years, more and more traditional Chinese medicine (TCM) have been reported to ameliorate the symptoms of AD, including Kai-Xin-San (KXS), which was first presented by Sun Simiao in the book of Bei Ji Qian Jin Yao Fang during the Tang dynasty, consisting of Polygala tenuifolia Willd (PR), Panax ginseng C.A. Mey (GR), Poria cocos (Schw.) Wolf (PO), and Acorus tatarinowii Schott (AT) [19]. As a classic formula, KXS has been applied in amnesia treatment for thousands of years [20] and displays profound therapeutic effect for dementia and depression in clinical trials[21]. Some researchers have found that KXS exerts cardioprotective effects in a myocardial infarction [22], and could ameliorate chronic fatigue syndrome by affecting the levels of inflammatory factors [23]. KXS has been reported to ameliorate cognitive dysfunction in AD animal model by increasing cholinergic and glutamatergic neurotransmission and promoting Aβ_42_ degradation [24–28]. However, as TCM formulas contain multiple chemical components, the specific pharmacological mechanisms through which KXS exert their effects against AD are still difficult to illustrate.

The network pharmacology is well suited for analyzing the multi-targeted agents, so network pharmacology methods may be appropriate for identifying the complex mechanisms of KXS. In the present study, we screened 30 targets of KXS associated with AD and predicted the mechanisms of KXS in the treatment of AD by network pharmacology. Then, we validate the predictions of bioinformatics in senescence-accelerated mouse prone 8 (SAMP8) mice, which was originally generated from AKR/J mice in the 1970s in the laboratory of Professor Takeda at Kyoto University in Japan [29]. SAMP8 mice is particularly well-suited to study the “transitional switch” between aging and AD as it exhibits spontaneous cognitive decline and Tau protein activation [30] found in aged individuals [31–33] and, to a greater extent, in patients with AD[34]. And most neurodegenerative alterations of SAMP8 mice emerged from 7 months old [35]. In this study, we chose 7-month-old SAMP8 mice as AD mouse model to be examined whether KXS could be inhibit neuronal apoptosis and improve learning and memory functions after 3-month (5.4 g/kg once per day, oral gavage) KXS treatment. Subsequently, the potential underlying action mechanisms of KXS on AD predicted by the network pharmacology analyses were experimentally validated in SAMP8 mice. We expect that our results can elucidate the regulatory mechanism of KXS therapeutic effects in AD and highlight the potential of KXS as a drug for ameliorating AD-like pathology and cognitive impairment.

## Materials and Methods

### Network Pharmacology Analysis

Chemical compound information was obtained from the Encyclopedia of Traditional Chinese Medicine (ETCM) database for all the 4 medicinal herbs (ETCM, http://www.nrc.ac.cn:9090/ETCM/) [26]. Target genes of TCM ingredients, herbs, and formulas were collected according to the chemical fingerprint similarity between TCM ingredients and known drugs. High correlation target of a drug-compound-target is defined by a QED score greater than 0.8 [36]. The DisGeNET database, AlzPlatform database, and GeneCards database were screened to collect the known AD-related targets. Cytoscape (version 3.6.1) was used to construct the active components and AD target (C-T) network of KXS. We constituted protein-protein interaction (PPI) networks by using the STRING network database platform to further elucidate the interactions of the common target genes in the treatment of AD with KXS. Gene ontology (GO) enrichment analysis was performed for identifying BP (biological process) of the potential targets of KXS for AD. Kyoto Encyclopedia of Genes and Genomes (KEGG) pathway enrichment analysis of the predicted targets can provide a more systematic and comprehensive understanding of the action mechanisms of the KXS on AD. In GO and KEGG analysis, *p* < 0.05 was defined as significantly enriched. Finally, significant pathways and its corresponding targets were imported into Cytoscape v3.6.1 to build a network diagram of the target-pathway(T-P).

### Preparation of KXS

All herbs were purchased from Anhui Yiyuantang Sinopharm Co., Ltd. (Anhui, China) and authenticated by Prof. Dong-Yu Min (Affiliated Hospital of Liaoning University of Traditional Chinese Medicine, Shenyang, China) as shown in Supplementary Table 1. The ratio of PR, GR, PO, and AT was 2:3:3:2. The four herbal materials of KXS were soaked in cold water for 2 h before being boiled for 40 min. The first decoction was obtained. Then, the four herbal materials were boiled a second time for 40 min to obtain the second decoction. Finally, the first and second decoctions were mixed, filtered through gauze, and concentrated followed brought to volume to obtain a final extract (0.54 g/mL). For chemical identification of KXS, HPLC chromatographic analysis was conducted. The HPLC analysis procedure and characteristic chromatogram of KXS are shown in Supplementary Fig. 1.

### Animals and Treatments

A total of 12 male SAMP8 mice (weight, 28–32 g; age, 7-months old) were used in the present study. The mice were housed in a specific pathogen-free (SPF) environment with a 12-h light/12-h dark cycle, allowed free access to water and standard food. All animals were treated according to the guidelines for the Care and Use of Laboratory Animals and the experimental procedures were approved by the Ethics Committee for Animal Use of China Medical University. The animals were randomly divided into vehicle group and KXS treatment group (6 mice/group). The KXS group was treated with KXS at a dosage of 5.4 g/kg body weight by oral gavage once daily for 3 months, and the vehicle group was treated with an equivalent volume of saline, and the anti-AD effect of KXS at such dosage has been proved in several previous studies [37, 38]. The dosage of KXS for the mice was based on clinical dose for human (weigh 70 kg) and then converted to mice dose according to the guidelines of FDA calculator based on the surface area of human and mice [39].

#### Behavioral Testing

After 3 months of KXS administration, mouse behavior was tested using Morris water maze (MWM) and the nest building tests, as described previously [40]. In general, mice were pretrained for 2 consecutive days in a circular water maze with a visible platform for MWM. The mice were expected to find the visible platform within 1 min; otherwise, mice were guided to find the platform and stayed on the platform for 15 s. After 2 days of training, the visible platform was hidden underneath 1 cm of water. Then, the mice were put into the water maze to find the hidden platform. This navigation test lasted for 5 days. The latency time and path length to find the hidden platform were recorded by the video-tracking SMART system version 3.0.06 (Panlab, Harvard Apparatus, MA, USA). In the probe trial on day 8, the platform was removed, and mice were allowed to swim for 1 min. The number of times that mice passed through the location of the platform was recorded.

In the nest building test, mice were singly housed. Eight pieces of paper in square shapes (5 $\times$ 5 cm) were placed in the cage. The nest was photographed for 7 consecutive days and scored as follows: 1—no nesting, 2—nesting partly, 3—majority shredded with no identifiable nesting site, 4—an identifiable nesting site but with a flat part within the nest, and 5—perfect nest.

#### Tissue Preparation

After behavior tests, mice were sacrificed, and the brains were removed immediately and placed on ice. Each brain was divided into two hemispheres along the midline. One-half was snap frozen for biochemical analysis, and the other was fixed in 4% paraformaldehyde for morphological analysis.

#### Western Blot Analysis

Mouse brain tissues were lysed with ice-cold RIPA buffer containing 1% protease inhibitor cocktail (Sigma-Aldrich, St. Louis, MO, USA; 8340) and 2% phosphatase inhibitors (Abcam, Cambridge, MA, USA; ab201112). The protein concentrations were measured by Bradford assay (Beyotime Institute of Biotechnology; P0010). Equal amounts of protein were separated on 10% SDS-PAGE gel and transferred onto PVDF membranes (Millipore, Billerica, MA, USA). Membranes were then incubated in 5% skim milk for 1 h and then incubated overnight at 4°C with the primary antibodies (Supplementary Table 2). Membranes were washed with TBS-T 3 times for 10 min each and then were incubated with horseradish peroxidase–labeled secondary antibodies (HRP 1:10000, Thermo Fisher Scientific, Waltham, MA, USA) for 1.5 h at room temperature. After washing 3 times, bands were detected using ECL (EMD Millipore). Protein band intensity was quantified using ImageJ software.

#### TNF-α ELISA

Mouse tumor necrosis factor (TNF-α) level was determined by ELISA kit (CUSABIO, China). Brain tissues were homogenized by sonication in 9 vol. of cold medium (Elabscience, China) and centrifuged for 5 min at 5000 *g*, at 4°C. The concentration of TNF-α in 100 μL samples was determined according to the manufacturer’s protocol, and the sample values were then obtained from the standard curve. Every sample was measured in duplicate. The OD value was measured at 450 nm in a microplate reader (Cytation5; BioTek, Winooski, VT, USA).

#### Immunohistochemistry

Tissue blocks from SAMP8 mouse brains were embedded in paraffin and coronally sectioned into 5-µm-thick slices, mounted onto gelatin-coated slides. Deparaffinization was performed with 2-h incubation at 60°C, followed by antigen retrieval in citric acid buffer. Immunohistochemistry was performed using a staining kit (MXB, Fuzhou, China). Briefly, the sections were quenched using 3% H_2_O_2_-methanol for 10 min. After washing and blocking with normal goat serum for 30 min, primary antibodies were applied (Supplementary Table 2) and sections were incubated overnight at 4°C. On the second day, after washing, biotinylated goat anti-rabbit IgG were applied and incubated for 2 h at room temperature followed by amplification with streptavidin peroxidase for 30 min. After incubation, the sections were developed in diaminobenzidine (Sigma-Aldrich, St. Louis, MO, USA) for coloration, and then immersed in distilled water to halt the reaction. The sections were then counterstained with hematoxylin and differentiated in hydrochloric acid alcohol. After dehydration, clearing, and mounting with neutral gum, the sections were observed under a light microscope (ECHO, San Diego, CA, USA). Blinded staining was performed and evaluated by two independent investigators.

#### Nissl Staining

Deparaffinization was performed with 2-h incubation at 60°C. According to the kit instructions, the procedure was conducted. The sections were treated with 0.1% cresyl violet at 56°C for 1 h. After differentiation for 2 min with Nissl differentiation solution, the stained sections were dehydrated with a graded series of ethanol solutions. Following clearing with xylene, the slices were mounted with neutral balsam and examined with a microscope. Blinded staining was performed by two independent investigators. Blinded staining was performed and evaluated by two independent investigators.

#### Statistical Analysis

All results were obtained from three independent experiments and presented as mean ± SEM. Differences between groups were evaluated by the unpaired two-tailed Student’s *t*-test. *p* < 0.05 was considered statistically significant.

## Results

### Network Pharmacology Predicts Potential Pharmacological Mechanisms for KXS Treatment of AD

**Fig. 1:**
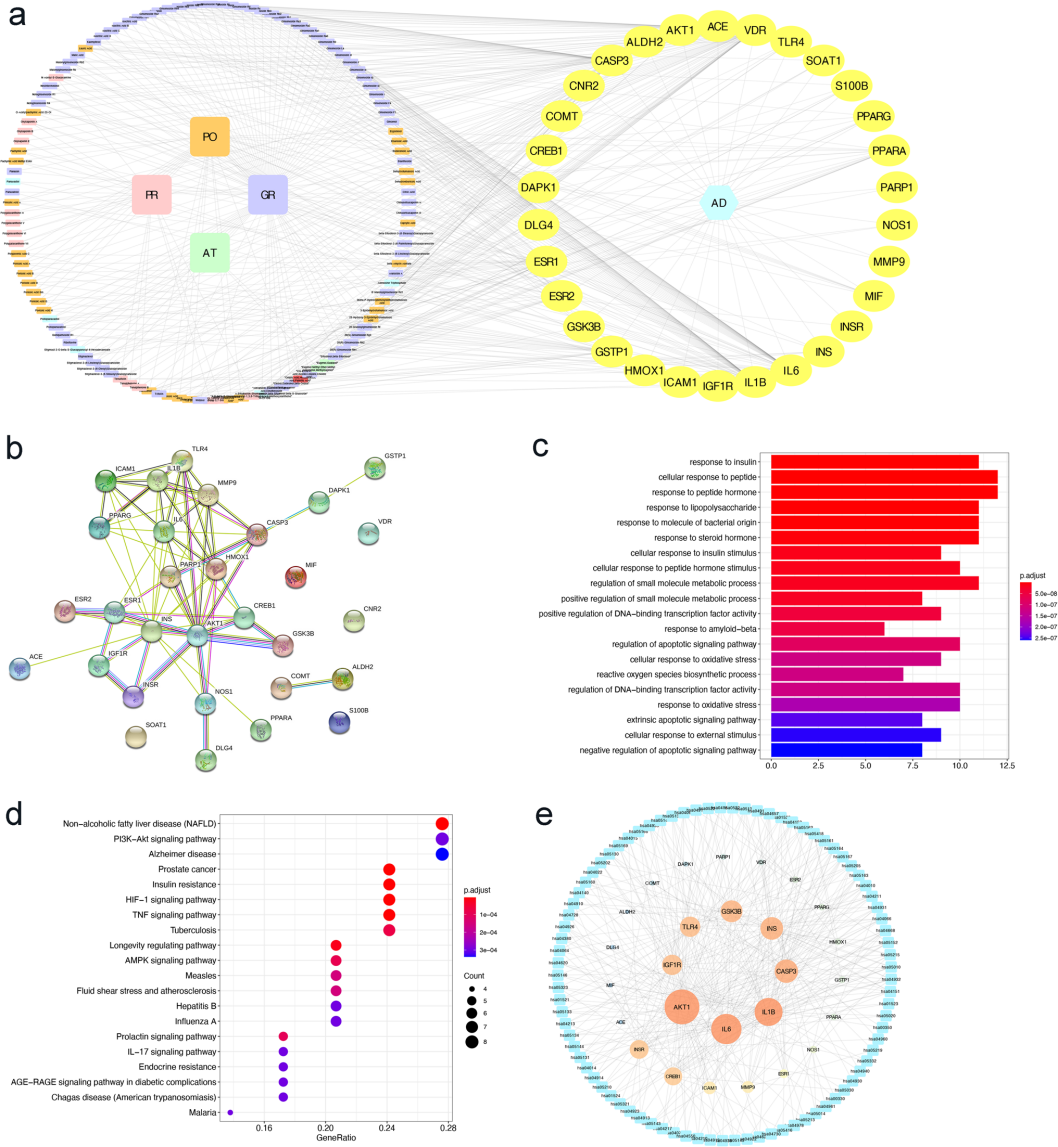
Network pharmacological prediction for KXS treatment of AD. **a** Compound-disease-target networks of KXS against AD. The rectangle nodes represent ingredients of herbs (orange for PO, purple for GR, green for AT, pink for PR, and red for Multidrugs-GR and PO), and the yellow ellipse nodes represent targets. **b** Protein-protein interactions identified by STRING software. **c** GO enrichment analysis of the intersecting targets’ biological process. **d** KEGG pathway enrichment analysis. **e** Target-pathway networks for KXS treatment of AD. The blue nodes represent the related pathways, and the ellipse nodes represent potential targets. The color depth and size of nodes are in proportion to their degree

In the present study, a total of 168 active compounds (including 76 in GR, 29 in PO, 25 in PR, 7 in AT and 2 compounds overlapped between GR and PO) of KXS were obtained from the ETCM online databases, corresponding to 863 prediction targets. These targets were further analyzed through the DisGeNet, AlzPlatform, and GeneCard databases to check if they were relevant with AD. Finally, 30 common targets of KXS in the treatment of AD were obtained. To facilitate visualization and further explanation of the target prediction results, we constructed the C-T network, which embodied 150 nodes (120 candidate compounds and 30 potential targets) and 364 compound-target interactions (Fig. 1a, Supplementary Table 3). The C-T network revealed that most compounds exerted multi-target effects, representing multifarious therapeutic effects. We further extracted the 30 significant targets to construct the PPI containing 30 nodes and 68 edges based on the STRING database, and the PPI enrichment *p*-value of these hub genes was 1.0 $\times$ 10^−16^, indicating that they were at least partially biologically connected (Fig. 1b).

The candidate targets in the PPI network were elucidated by performing GO and KEGG pathway enrichment analysis. The top 20 items enriched in the BP category are shown in Fig. 1c. The results of GO enrichment analysis could be classified into some functional modules related with AD-associated pathological processes, including anti-inflammatory, anti-apoptotic, and antioxidant effects. Next, we performed KEGG pathway enrichment analysis to examine the pathways for putative therapeutic targets of KXS for AD. The top 20 pathways were determined (Fig. 1d), mainly including pathways in non-alcoholic fatty liver disease (NAFLD), PI3K-AKT signaling pathway, AD, and other dysregulation of predominantly inflammatory signaling pathways. All the targets interacting with the active ingredients were mapped onto the 85 KEGG pathways, and the T-P network was generated. As shown in Fig. 1e, the AKT target shows the highest number of pathway connection, followed by interleukin-6 (IL-6), interleukin-1 beta (IL-1β), caspase 3, Insulin (INS), GSK3β, Toll-like receptor 4 (TLR4), and type 1 insulin-like growth factor receptor (IGF1R). The details are provided in Supplementary Table 4. Moreover, molecular docking results indicated that Kaempferol, Onjisaponin A, Ginsenoside Ii, Chikusetsusapon, Ginsenoside F1, and Cis-9, Cis-12-Linoleic-Acid exhibited good affinity for AKT, IL-6, IL-1β, caspase 3, and TLR4, respectively. The details are provided in Supplementary Fig. 2.

### KXS Attenuates Tau Hyperphosphorylation by Suppressing GSK3β and CDK5 Protein Kinase Activation in SAMP8 Mice

**Fig. 2:**
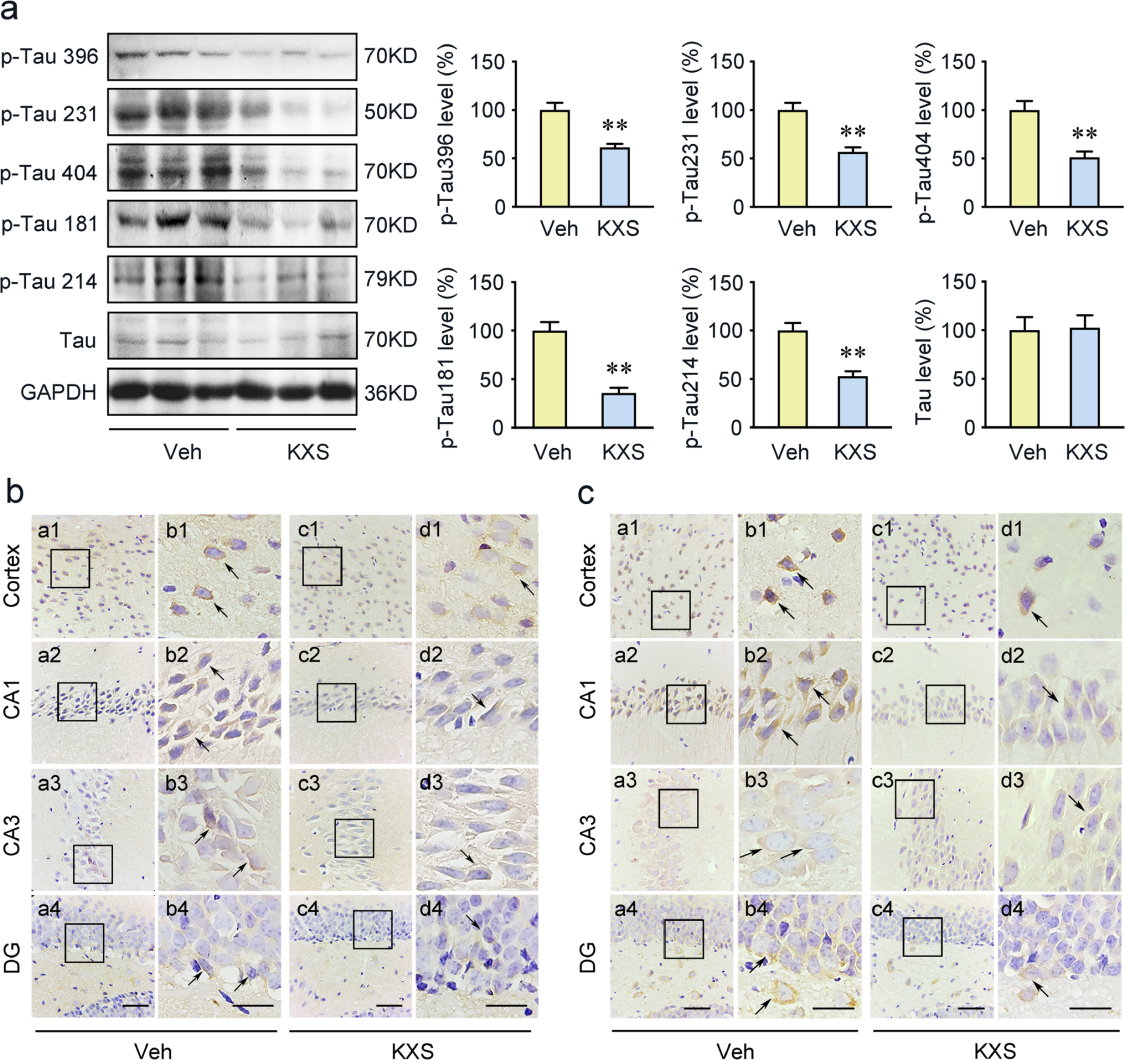
KXS abrogates Tau hyperphosphorylation in SAMP8 mice. **a** The levels of Tau phosphorylation at Ser396, Thr231, Ser404, Thr181, and Ser214 proteins were examined by Western blot assays. Tau phosphorylation at Ser404 (**b**) and Thr231 (**c**) immunohistochemical staining in cerebral cortex, CA1, CA3, and DG regions with the vehicle (Veh) group and KXS group. Right panels of each group show the representative images in high magnification. Arrows indicate the phosphorylation Tau positive staining. Values were the mean ± SEM (*n* = 5–6). *p < 0.05; **p < 0.01 versus the vehicle group. Scale bars: 60 μm. Scale bar is 30 μm in the high magnification of right panels

Based on the results of network pharmacology, GSK3β is AD-associated targets of KXS and the kinase responsible for Tau hyperphosphorylation [41]. Tau hyperphosphorylation is a predominant pathological hallmark of AD, and it contributes to AD development by causing synaptic impairments, neuronal dysfunction, and NFTs formation [42]. Therefore, we employed immunohistochemistry and Western blot to determine whether KXS treatment could regulate Tau phosphorylation through GSK3β signaling pathway. We investigated the Tau phosphorylation level at multiple sites by Western blot. We observed decreased Tau phosphorylation levels at Ser396, Thr231, Ser404, Thr181, and Ser214 in the KXS group (Fig. 2a). The staining results further suggested that KXS treatment significantly inhibited Tau hyperphosphorylation at both Ser404 and Thr231 sites in cerebral cortex, CA1, CA3, and DG regions (Fig. 2b and c). The phosphorylated Tau-positive cells were shown as brown granules, mainly expressed in the cytoplasm mostly with a perinuclear pattern.

**Fig. 3:**
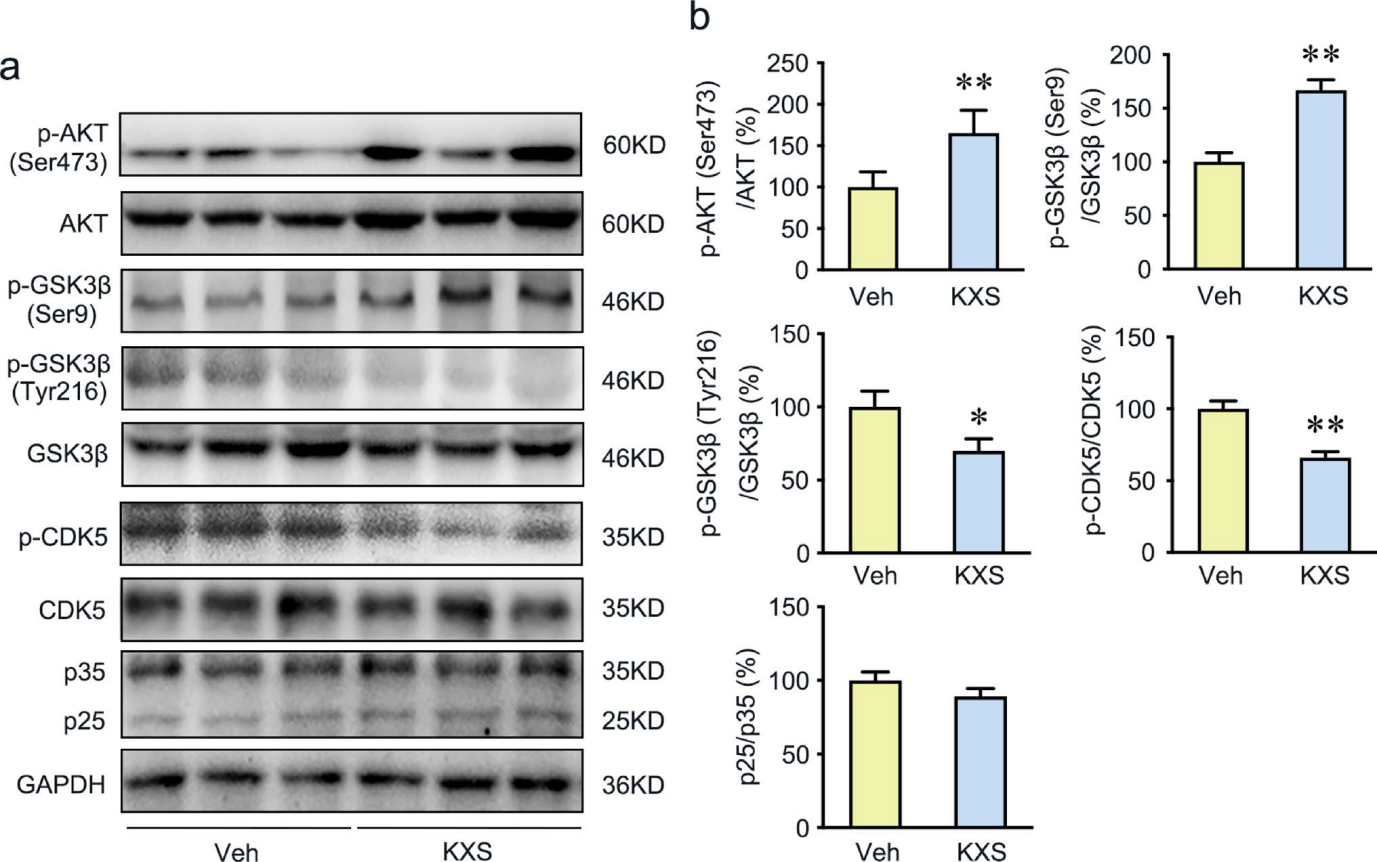
KXS represses Tau hyperphosphorylation by suppressing the AKT/GSK3β signaling and CDK5 protein kinases. **a** Western blot analysis of p-AKT (Ser473), AKT, p-GSK3β (Ser9), p-GSK3β (Tyr216), GSK3β, p-CDK5 (Tyr15), CDK5, p35, and p25 expression in the brains of vehicle group and KXS group mice. **b** Quantified protein expression levels of above protein. Values were the mean ± SEM (*n* = 5–6). *p < 0.05; **p < 0.01 versus the vehicle group

GSK3β, CDK5, and P35/25 are key participants of Tau hyperphosphorylation. Thus, we further evaluated these kinase expression levels by Western blot. The significantly decreased p-GSK3β (Tyr216)/GSK3β ratio and increased p-GSK3β (Ser9)/GSK3β ratio were observed in KXS group (Fig. 3), which were consistent with the network pharmacology results. In addition to GSK3β, p-CDK5 protein expression in SAMP8 mice brains was also significantly repressed when compared to vehicle group (Fig. 3). In contrast, the p35 and p25 expression levels were not significantly different between the vehicle and KXS groups (Fig. 3). Furthermore, p-Akt (Ser473) phosphorylates GSK3β to inhibit its activity. And we have detected the expression of p-AKT(Ser473), AKT in brains from both groups. The significantly increased p-AKT(S473)/AKT ratio was observed in KXS group. The results indicate that KXS inactivates GSK3β by an increase in p-Akt (Ser473). All the results indicate that KXS may inhibit Tau hyperphosphorylation by suppressing the activation of GSK3β and CDK5.

### KXS Inhibits Neuroinflammation in the Brain of SAMP8 Mice

**Fig. 4:**
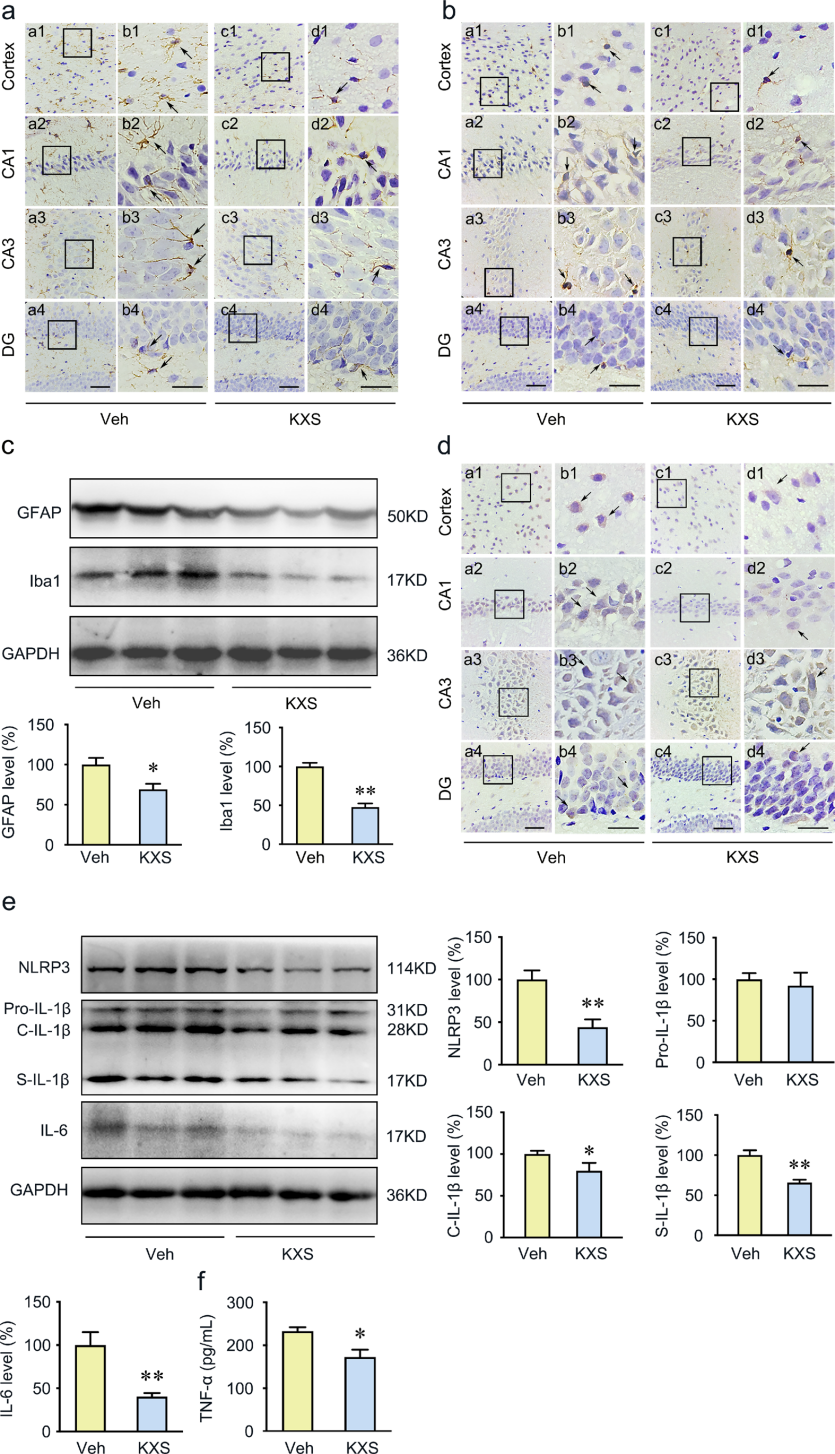
KXS inhibits neuroinflammation in the brain of SAMP8 mice. **a**, **b** Astrocytes were labeled with anti-GFAP antibodies; microglia were labeled with anti-Iba1 antibodies. Right panels of each group show the representative images in high magnification. Arrows point to GFAP or Iba1 positive cells. **c** Western blot analysis of GFAP and Iba1, and quantification, in the SAMP8 mice brain. **d** Representative immunohistochemistry images showing the labeling of NLRP3 within the cerebral cortex and CA1, CA3, and DG region areas of the hippocampus from the brain slices of vehicle group and KXS group mice. Right panels of each group show the representative images in high magnification. The arrow indicates the NLRP3 positive cells. **e** Western blot analysis revealed the protein expression of NLRP3, IL‐1β, and IL-6 in the brains of vehicle group and KXS group mice. **f** Inflammatory factors TNF-α detected by ELISA. Values were the mean ± SEM (*n* = 5–6). *p < 0.05; **p < 0.01 versus the vehicle group. Scale bars: 60 μm. Scale bar is 30 μm in the high magnification of right panels

The network pharmacology analysis indicated that the therapeutic mechanism of KXS for AD involves neuroinflammation. To investigate whether KXS prevents inflammatory response, we evaluated the astrocytic and microglial activities by detecting the expression of anti-GFAP and anti-Iba1 antibodies in the hippocampus and cortex of SAMP8 mice. Arrows indicate the GFAP or Iba1-positive cells. We observed that the expression of GFAP and Iba1 following KXS treatment significantly reduced compared to the vehicle mice (Fig. 4a and b). We also measured GFAP and Iba1 levels using Western blot, and the results were consistent with immunochemistry (Fig. 4c).

Considering the pathogenic role of inflammation in the development of AD, the expression levels of several important inflammatory factors were further evaluated by immunohistochemistry, Western blot, and ELISA, including nucleotide-binding oligomerization domain-like receptor family, pyrin domain containing 3 (NLRP3), IL-1β, IL-6, and TNF-α. The results showed KXS treatment significantly decreased NLRP3 inflammasome protein expression (Fig. 4d and e). The assembled NLRP3 inflammasome is responsible for the cleavage of IL-1β into secreted form [43]. Therefore, in the present study, we found that KXS repressed the secretion of IL-1β (Fig. 4e). Moreover, IL-6 and TNF-α were downregulated in the KXS group compared to the vehicle group (Fig. 4e and f). These results suggest that KXS effectively attenuates microglia and astrocyte activation, and effectively suppress the inflammatory cytokine secretion.

### KXS Inhibits TLR4/MyD88/NF-κB Signaling Pathway in SAMP8 Mice Brain

**Fig. 5:**
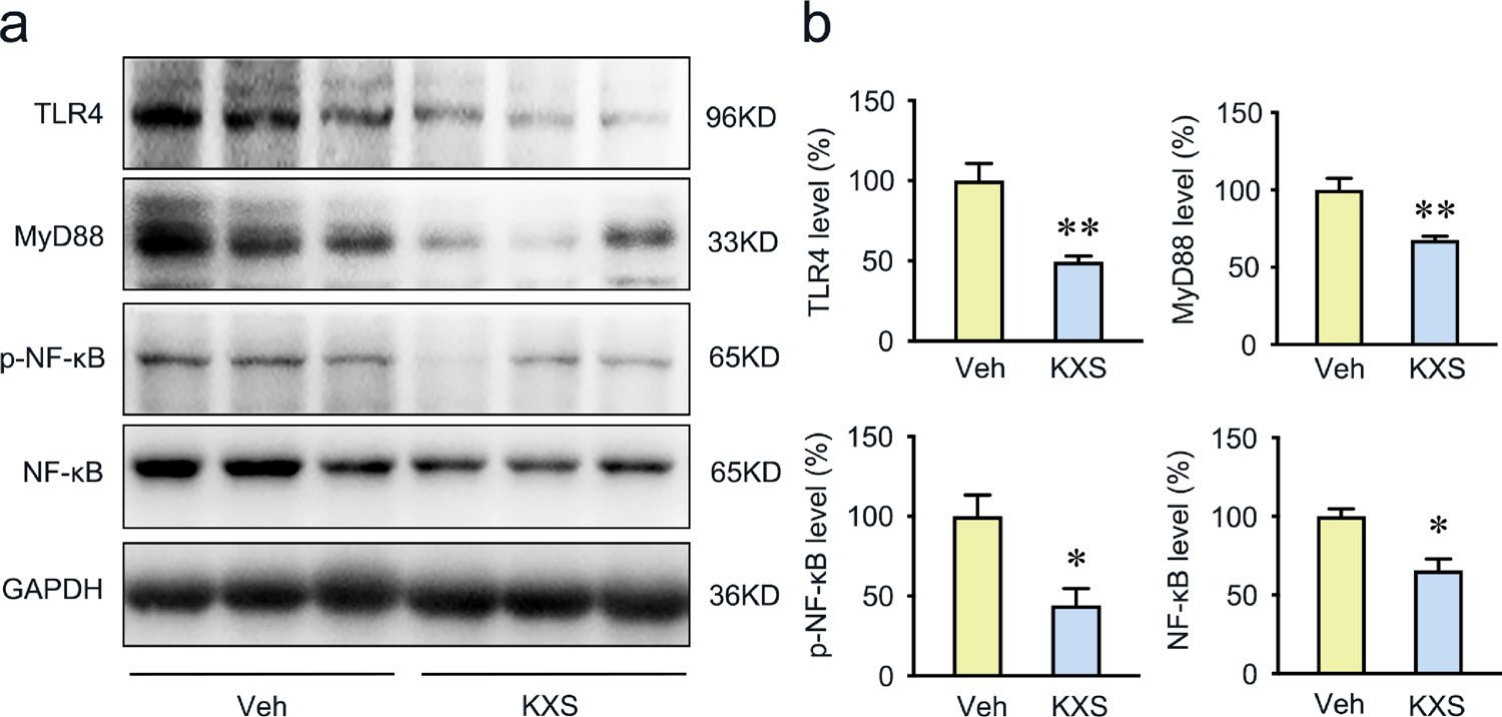
KXS inhibits TLR4/MyD88/NF-κB signaling pathway in SAMP8 mice brain. **a** Western blot analysis of TLR4, MyD88, NF-κB subunit p65, and NF-κB subunit p65 phosphorylation on ser536. **b** Quantitative analysis of TLR4, MyD88, NF-κB, and p-NF-κB protein expression. Values were the mean ± SEM (*n* = 5–6). *p < 0.05; **p < 0.01 versus the vehicle group

Based on the network pharmacology results, TLR4 and its downstream proteins, myeloid differentiation factor 88 (MyD88) and nuclear factor-kappa B (NF-κB), were selected to further explore the effect of KXS on the neuroinflammation pathway. Our results demonstrated that KXS significantly decreased the expression of TLR4 (Fig. 5). As the known downstream effector of TLR4, MyD88 and NF-κB are critical for TLR4-mediated inflammation; therefore, we determined their expression level by Western blot. As shown in Fig. 5, the protein expression of MyD88 was significantly decreased by KXS treatment. Besides, we also observed a significant reduction in p-NF-κB (Ser536) and NF-κB expression in KXS treatment group (Fig. 5). Taken together, the results indicate that KXS attenuates neuroinflammation in SAMP8 mice by inhibiting the TLR4/MyD88/NF-κB signaling pathway.

### KXS Attenuates Apoptosis in SAMP8 Mice

**Fig. 6:**
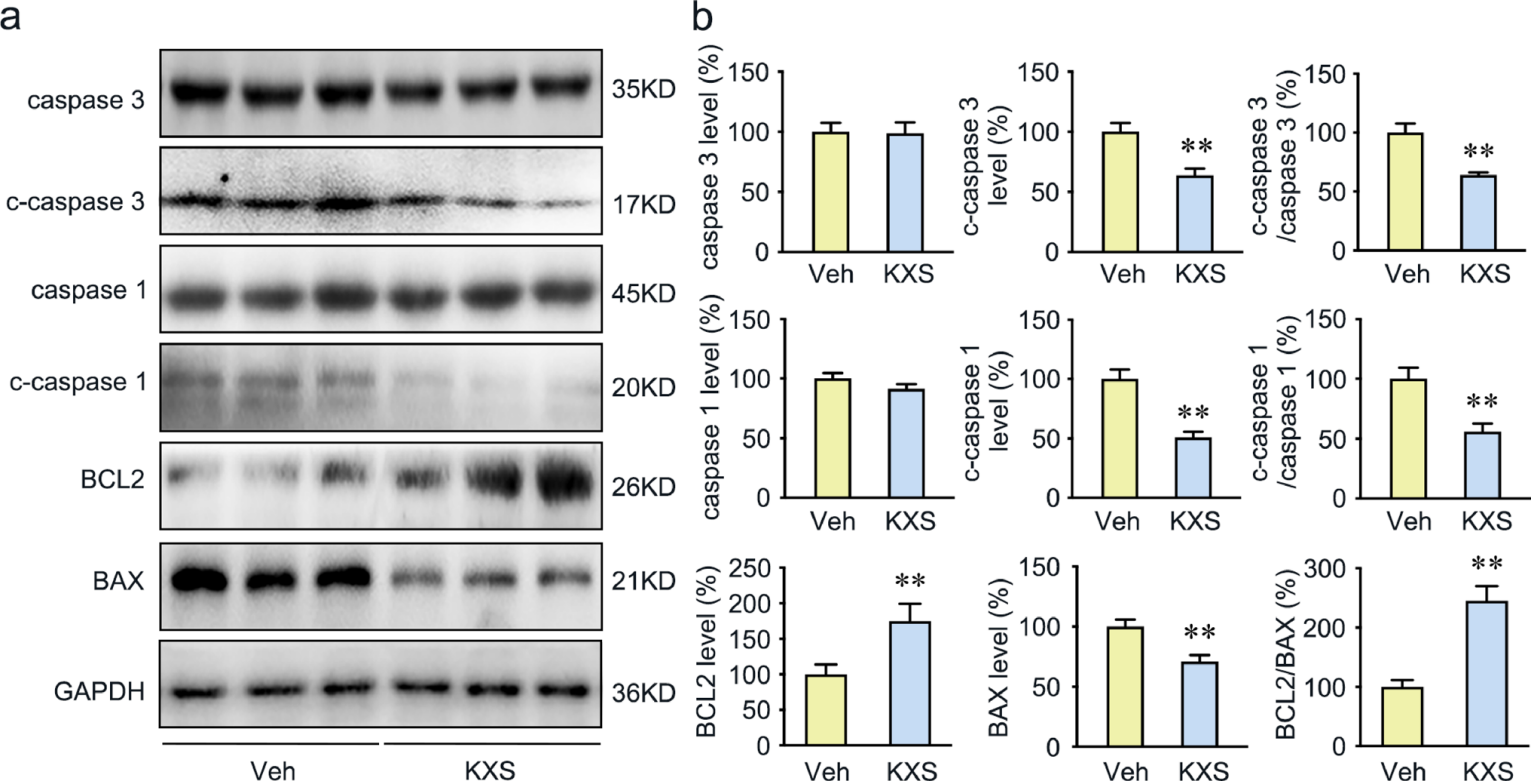
Protective effect of KXS against apoptosis in SAMP8 mice. **a** Western blot analysis of caspase 3, cleaved caspase 3 (c-caspase 3), caspase 1, cleaved caspase 1 (c-caspase 1), BCL2, and BAX. **b** Quantitative analysis of caspase 3, cleaved caspase 3, c-caspase 3/ caspase 3 ratio, caspase 1, cleaved caspase 1, c-caspase 1/caspase 1 ratio, BCL2, BAX, and BCL2/BAX ratio. Values were the mean ± SEM (*n* = 5–6). *p < 0.05; **p < 0.01 versus the vehicle group

Our network pharmacology analysis suggested that KXS could regulate the expression of the target protein caspase 3, which is the critical effector of apoptosis. To verify whether KXS participates in the regulation of apoptosis, several apoptosis-related proteins were determined by Western blot analysis. A significant decrease in the cleaved-caspase 3 and the cleaved-caspase 1 level was observed in the KXS-treated group (Fig. 6). Besides, KXS enhanced B cell lymphoma 2 (BCL2) expression and suppressed BCL2-associated X protein (BAX) expression, resulting in upregulation of BCL2/BAX ratio. Nissl stains were performed to assess the neuroprotective functions of KXS on the mouse brain. As shown in Supplementary Fig. 3, compared with KXS-treated SAMP8 mice, the vehicle group exhibited significant nuclear breakdown and less intact Nissl substance in the cortex and hippocampus. All the results suggest that KXS attenuates neuronal apoptosis by decreasing the BCL2/BAX ratio, inhibiting caspase 1 and caspase 3 activity in SAMP8 mice.

### KXS Ameliorates Memory Deficits in Aged SAMP8 Mice

**Fig. 7:**
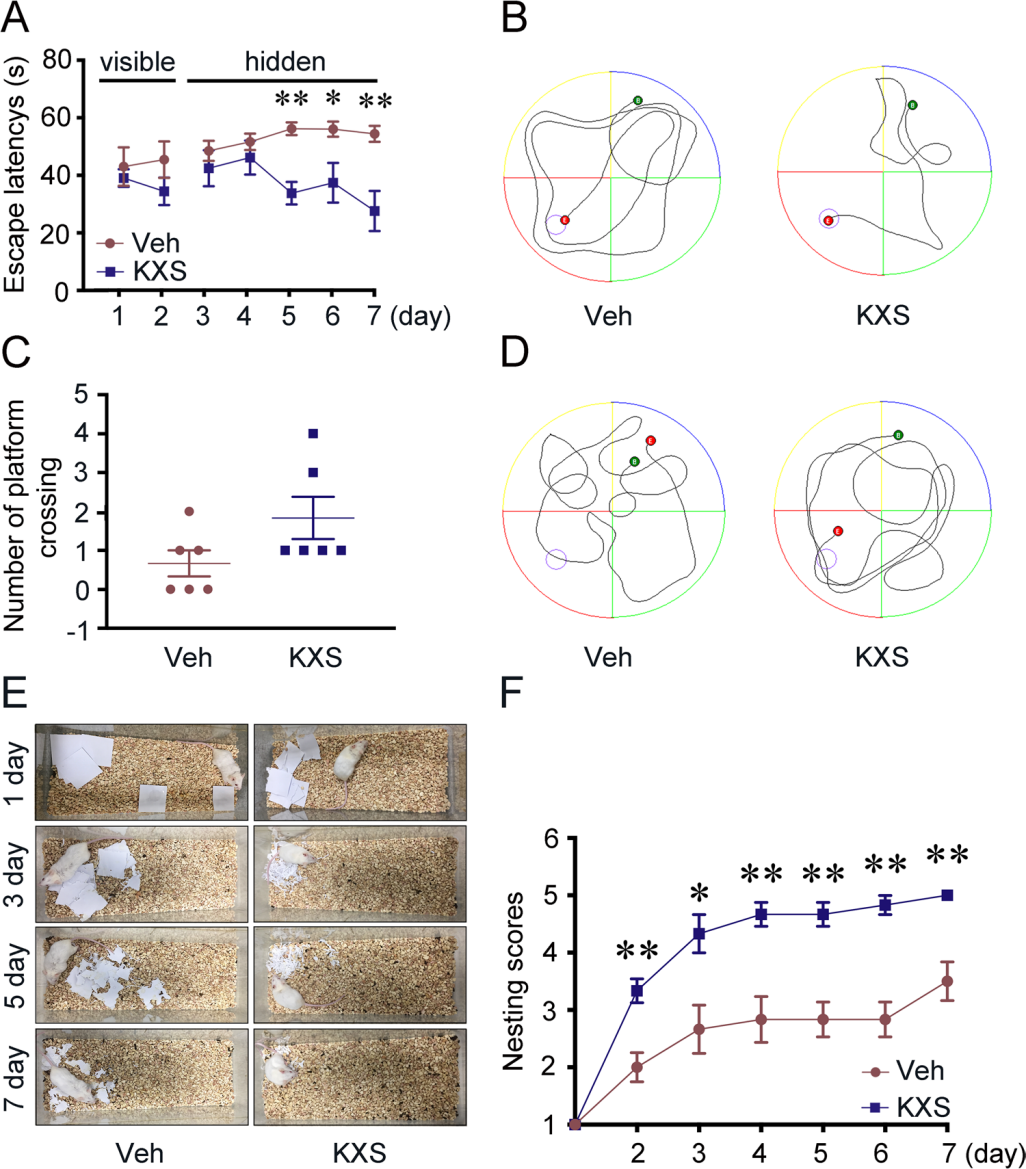
KXS treatment ameliorates the spatial learning and reference memory deficiency of SAMP8 mice. **a** Escape latencies in visible and hidden platform during MWM task training in both the vehicle group and KXS group. **b** Movement track of both groups in in the hidden platform trail. **c** Number of crossing the platform location of each group in the probe test. **d** Movement track of both groups in the probe trail. **e** Representative images were obtained from 0 to 7days in the nesting behavior test. **f** Nest-building scores for each group. Values were the mean ± SEM (*n* = 6). *p < 0.05; **p < 0.01 versus the vehicle group

To investigate whether KXS treatment improved cognitive function in SAMP8 mice, MWM and nest building tests were performed. In the visible-platform tests of MWM, there were no significant differences between vehicle and KXS-treated groups (Fig. 7a), indicating that KXS administration did not influence the motility or vision of the mice. However, KXS-treated SAMP8 mice exhibited significantly shorter escape latency and travel distance to the platform than the vehicle group on days 5, 6, and 7, as shown in Fig. 7a and b. And both groups had similar swimming speed (Supplementary Fig. 4), which suggests that the differences in latency time between groups are not due to the variation in motoric abilities. All the results indicating that KXS could markedly improve the spatial memory of SAMP8 mice. In the probe trial, KXS-treated mice exhibited no significant differences compared to the vehicle mice, with the number of platform location crossings of KXS group showing an increasing trend (Fig. 7c and d).

We further assessed the effects of KXS on general health and hippocampal function with the nest building test. Treatment with KXS resulted in significant improvements in nesting scores, when compared to the vehicle group (Fig. 7e). Figure 7f shows the representative results in nest building test. Taken together, the results suggested that KXS improves cognitive impairment in SAMP8 mice.

## Discussion

Previous studies have shown that KXS, a classic formula for amnesia, ameliorated the pathological changes and improved cognitive function in animal models of AD [44, 45]. However, the bioactive ingredients of KXS and the drug targets are still not clear, which is unfavorable to KXS modern development and clinical utility. In the present study, we used network pharmacology to identify the bioactive ingredients and action mechanism of KXS in the treatment of AD. Since network pharmacology was based on computational analysis only, further verification is needed to validate the therapeutic mechanism of KXS. Therefore, a series of experiments were carried out to validate the predicted molecular mechanisms obtained from network pharmacology analysis. Network pharmacological analysis revealed a total of 168 active compounds and 863 targets in the KXS formula. We further identified 30 targets closely correlated with AD through Venn diagram. Based on the aforementioned GO and KEGG pathway enrichment analysis, an integrated AD pathway can be separated into four representative therapeutic modules, namely, Tau hyperphosphorylated module, inflammation module, cell apoptosis module, and oxidative stress module. Among relevant protein targets with the 85 related pathways, AKT, IL-6, IL-1β, caspase 3, INS, GSK3β, TLR4, and IGF1R were the key target nodes with the top degree, indicating that they may be the most likely targets for AD therapy of KXS. Previous research has found that KXS could improve learning and memory impairment in AD animal model by inhibiting oxidative stress [44, 46]. To investigate the mechanisms of the regulatory function of KXS during AD pathogenesis, in addition to antioxidant activities, we also wondered whether KXS influences the other modules.

Network pharmacology analysis suggested AKT and GSK3β were the important therapeutic target of KXS. GSK3β is the key kinases for Tau phosphorylation. The activation of GSK3β could be inhibited by phosphorylation at Ser9 or promoted by phosphorylation at Tyr216. Overactivity or overexpression of GSK3β increases the phosphorylation of most serine and threonine residues of Tau, which leads to Tau aggregation and neuronal dysfunction in AD [47–50]. In line with the predicted results of network pharmacology, we detected a remarkably decreased level of GSK3β (Tyr216) and increased level GSK3β (Ser9) of in KXS-treated group, which indicated a significant inhibition of GSK3β activation. The best studied mechanism by which GSK3β is regulated is Akt-dependent serine phosphorylation [51]. AKT phosphorylates GSK3β at the S9 residue, which inactivates GSK3β [52]. And the phosphorylation of Akt (Ser473) is the primary phosphorylated form after the activation of Akt[53]. As a result, we examined Ser473 phosphorylation of Akt in this study. KXS significantly increased p-AKT(S473) expression, which indicates that KXS prevent tau phosphorylation via Akt/GSK3β signaling because Akt activation inhibits tau phosphorylation through GSK3β inactivation. In addition, we also evaluated p-CDK5(Tyr15), CDK5, p35, and p25 levels after KXS treatment. CDK5 appears to be key factor in Tau phosphorylation, and the activation of CDK5 is significantly increased in AD [53, 54]. Studies show that the CDK5 can be activated by phosphorylation at Tyr15 residues [54, 55] and the binding of p25 (the truncated form of p35) [56]. Our results showed that KXS remarkably reduced the level of p-CDK5 at Tyr15 sites, while the levels of CDK5, p35, and p25 were not changed. Consistently, the phosphorylation of Tau at several AD-related sites (Ser396, Thr231, Ser404, Thr181, and Ser214) was significantly reduced in both the hippocampus and cortex of SAMP8 mice by KXS treatment. Taken together, all these imply that KXS exerts inhibited function on Tau hyperphosphorylation in SAMP8 mice via predicted targets AKT/GSK3β and CDK5.

The network pharmacology analysis has identified KXS could modulate neuroinflammation through regulating IL-6, IL-1β, and TLR4. It is reported that the inflammatory processes during the pathogenesis of AD are mostly mediated by microglia and astrocytes [57]. Tau pathology is also regulated by neuroinflammations [58]. Therefore, we examined the activation of astrocytes and microglia by evaluating GFAP and Iba-1 level, which have been reported to be upregulated in the activated astrocytes and microglia in AD [59]. We found that KXS reduced significantly GFAP and Iba1 expression levels in the brain of SAMP8 mice, which indicated that KXS has a significant anti-inflammatory activity. Additionally, crosstalk between TLR4 and NLRP3 inflammasome promotes neuroinflammation in AD [60]. TLR4 is a transmembrane protein belonging to the pattern recognition receptor family [61], which is regarded as neuroinflammatory receptors detected in neurons, astrocytes, and microglia [62, 63]. MyD88 is an adaptor protein downstream of most TLRs and leads to the activation of NF-κB [64]. Once activated, the TLR4/MyD88/NF-κB signaling would initiate the NLRP3 inflammasome activation [65] and other cytokines (especially IL-6 and TNF-α) production [66, 67], which are considered as the key contributor to neuroinflammation during neurodegeneration. NLRP3 inflammasome is a multimeric protein that consists of NLRP3, adaptor protein ASC, and pro-caspase 1 [68, 69]. The activation of NLRP3 inflammasome leads to the cleavage of pro-caspase 1 into active caspase 1, which further cleaves pro-IL-1β into mature forms, and eventually drives the Tau pathology in AD brain [70, 71]. In our present study, we detected significantly reduced TLR4, MyD88, and NF-κB protein expression levels in KXS-treated mice. Consistent with the results, we also found KXS effectively suppressed activation of the NLRP3 inflammasome in SAMP8 mice. Pro-inflammatory elements such as IL-6, TNF-α, and IL-1β ultimately exacerbate neuroinflammation by stimulating the synthesis of Aβ and phosphorylation of Tau [72–74]. As expected, we also detected a decreased expression level of IL-6, TNF-α, and IL-1β in KXS-treated SAMP8 mice, which is consistent with the findings from other network pharmacology studies [75]. Our study indicates that KXS might exert inhibitory activities against neuroinflammation by suppressing NLRP3 inflammasome activation and pro-inflammatory cytokine (IL-6, TNF-α, and IL-1β) production through TLR4/MyD88/NF-kB signaling pathway. Our work complements and extends other studies on KXS using network pharmacology.

**Fig. 8:**
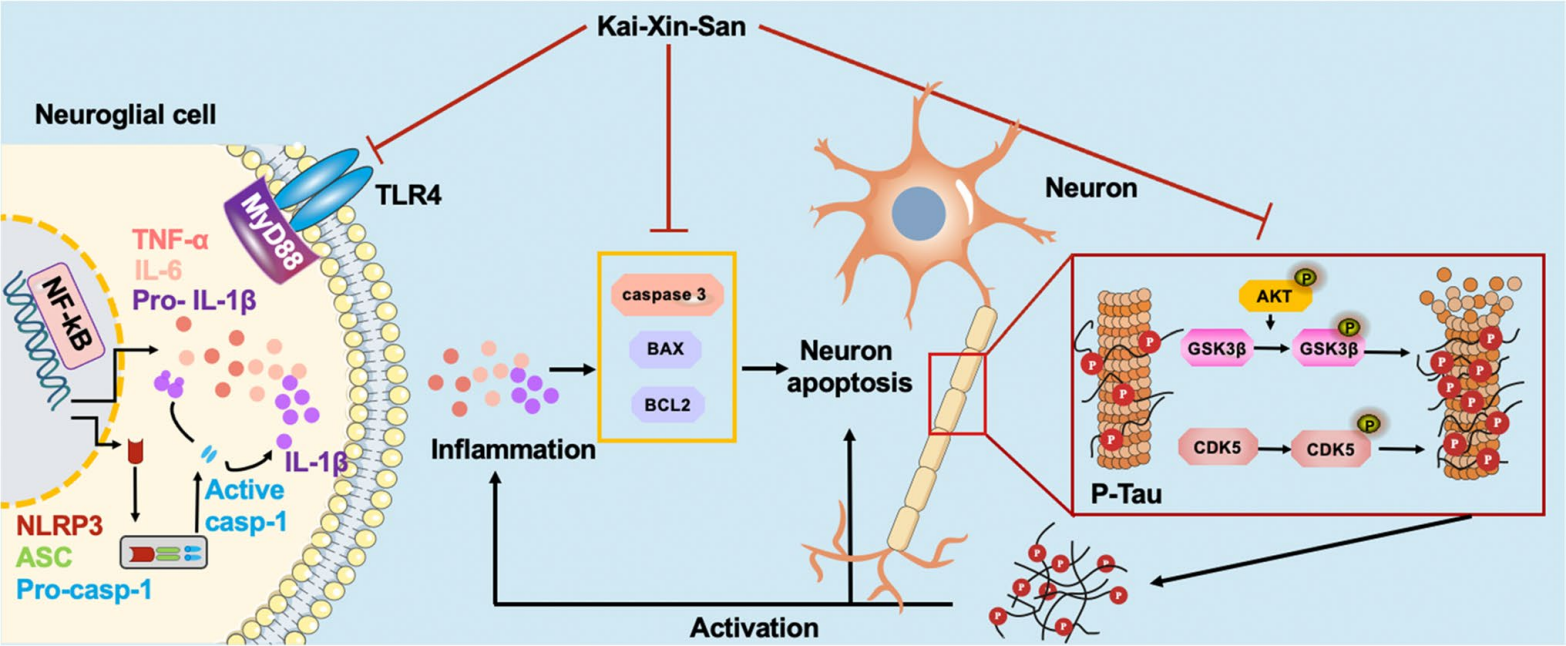
Diagram of the mechanism of action of KXS against AD pathogenesis

Hyperphosphorylated Tau and neuroinflammation contribute to the neuronal apoptosis in AD. In the present study, we demonstrated that KXS has remarkable inhibitory activity on Tau hyperphosphorylation and inflammation; joined with the network pharmacology analysis, we speculate that KXS may have anti-apoptosis activity. Previous studies have shown that caspase 1 activation, caspase 3 activation, and BCL2/BAX interference are directly involved in apoptosis [76, 77]. BCL2 exerts inhibitory effects on apoptosis while BAX exerts pro-apoptotic effects [78–81]. Caspase 3 is the key effector of apoptosis, the activation of caspase 3 would initiate irreversible apoptosis [82]. Caspase 1 activation induces the maturation and production of IL-1β and subsequently potentiates inflammatory responses [83, 84]. Proinflammatory cytokines such as IL-1β and TNF-α could also cause neuronal apoptosis via neurotoxic effect [85]. Our results showed that KXS may upregulate BCL2/BAX ratio, inhibit caspase 1 and caspase 3 activity, and alleviate apoptosis in the SAMP8 mice brain. Based on our finding, an overview of mechanism diagram for the neuroprotective effect of KXS against AD pathogenesis is shown in Fig. 8.

In light of the inhibitory role of KXS on Tau hyperphosphorylation, neuroinflammation, and apoptosis, we also considered the cognitive function of the KXS-treated mice. We observed that KXS significantly improved cognitive decline of SAMP8 mice in MWM and nest building test. Furthermore, INS and IGF1R, predicted targets of KXS in AD therapy, are also associated with diabetes. Thus, KXS may be beneficial for slowing the progression of AD through improving insulin resistance which requires further investigation in the future. We also found Kaempferol, Chikusetsusapon, Ginsenoside F1, Ginsenoside Ii, Onjisaponin A, Cis-9, and Cis-12-Linoleic-Acid were the core genes targeted by KXS compounds. These results suggested that KXS exerts therapeutic efficacy through the synergistic effect of multi-compounds, multi-targets, and multi-pathways. It is noteworthy that the multiple compounds of KXS may provide a mutual enhancement effect on AD treatment, but this must be further tested using single or mixed compounds.

## Conclusion

In this work, we combined network pharmacology approach and experimental validation in vivo to explore and verify the mechanisms of KXS’s potential in treating AD. We found that KXS upregulated AKT phosphorylation, suppressed GSK3β and CDK5 activation, and inhibited the TLR4/MyD88/NF-κB signaling pathway to attenuate Tau hyperphosphorylation and neuroinflammation, therefore suppressing neuronal apoptosis and improving the cognitive function of SAMP8 mice. Our present study clearly highlights the therapeutic value of KXS for attenuating the progression of AD. Furthermore, our study sheds novel lights on investigating the mechanisms of the TCM formula in AD therapy from the perspectives of bioinformatic methods. Our study is crucial for the future development of TCM modernization, including KXS, giving hopes for their further and broader clinical application.

## Supplementary Information

Below is the link to the electronic supplementary material.

Supplementary file1 (DOCX 2743 KB)

Supplementary file2 (XLSX 9.45 KB)

Supplementary file3 (XLSX 10.5 KB)

Supplementary file4 (XLSX 13.2 KB)

Supplementary file5 (XLSX 13.1 KB)

## Data Availability

The datasets generated and/or analyzed during the current study are available from the corresponding author on reasonable request. This article does not contain any studies with human participants performed by any of the authors.

## References

[1] International, A. s. D. (2019). World Alzheimer Report 2019: attitudes to dementia, Alzheimer’s Disease Internationals London. https://www.alzint.org/resource/world-alzheimer-report-2019/. Accessed 20 Sept 2019

[2] Park JS, Lee J, Jung ES, Kim MH, Kim IB, Son H, Kim S, Kim S, Park YM, Mook-Jung I, Yu SJ, Lee JH (2019). Brain somatic mutations observed in Alzheimer's disease associated with aging and dysregulation of tau phosphorylation. Nat Commun.

[3] Lee V-Y, Goedert M, Trojanowski JQ (2001). Neurodegenerative tauopathies. Annu Rev Neurosci.

[4] Iqbal K, Liu F, Gong CX, Grundke-Iqbal I (2010). Tau in Alzheimer disease and related tauopathies. Curr Alzheimer Res.

[5] Congdon EE, Sigurdsson EM (2018). Tau-targeting therapies for Alzheimer disease. Nat Rev Neurol.

[6] Lim H, Lee D, Choi WK, Choi SJ, Oh W, Kim DH (2020). Galectin-3 secreted by human umbilical cord blood-derived mesenchymal stem cells reduces aberrant tau phosphorylation in an Alzheimer disease model. Stem cells international.

[7] Lee HJ, Jung YH, Oh JY, Choi GE, Chae CW, Kim JS, Lim JR, Kim SY, Lee SJ, Seong JK, Han HJ (2019). BICD1 mediates HIF1α nuclear translocation in mesenchymal stem cells during hypoxia adaptation. Cell Death Differ.

[8] Xue Q, Yu C, Wang Y, Liu L, Zhang K, Fang C, Liu F, Bian G, Song B, Yang A, Ju G, Wang J (2016). miR-9 and miR-124 synergistically affect regulation of dendritic branching via the AKT/GSK3β pathway by targeting Rap2a. Sci Rep.

[9] Griñán-Ferré C, Vasilopoulou F, Abás S, Rodríguez-Arévalo S, Bagán A, Sureda FX, Pérez B, Callado LF, García-Sevilla JA, García-Fuster MJ, Escolano C, Pallàs M (2019). Behavioral and cognitive improvement induced by novel imidazoline I2 receptor ligands in female SAMP8 mice. Neurotherapeutics : the journal of the American Society for Experimental NeuroTherapeutics.

[10] Dhavan R, Tsai LH (2001). A decade of CDK5. Nat Rev Mol Cell Biol.

[11] Knezevic D, Mizrahi R (2017). Molecular imaging of neuroinflammation in Alzheimer’s disease and mild cognitive impairment. Prog Neuropsychopharmacol Biol Psychiatry.

[12] Lagarde J, Sarazin M, Bottlaender M (2018). In vivo PET imaging of neuroinflammation in Alzheimer’s disease. J Neural Transm.

[13] Barron MR, Gartlon J, Dawson LA, Atkinson PJ, Pardon MC (2020). Increasing tau 4R tau levels exacerbates hippocampal tau hyperphosphorylation in the hTau model of tauopathy but also tau dephosphorylation following acute systemic inflammation. Front Immunol.

[14] Azam, F., N.H. Alabdullah, H.M. Ehmedat, A.R. Abulifa, I. Taban, and S. Upadhyayula (2018) NSAIDs as potential treatment option for preventing amyloid β toxicity in Alzheimer’s disease: an investigation by docking, molecular dynamics, and DFT studies. Journal of Biomolecular Structure & Dynamics: p. 1-19. 10.1080/07391102.2017.1338164 10.1080/07391102.2017.1338164 28571516

[15] Zhao H, Wang Q, Cheng X, Li X, Li N, Liu T, Li J, Yang Q, Dong R, Zhang Y, Zhang L (2018). Inhibitive effect of resveratrol on the inflammation in cultured astrocytes and microglia induced by Abeta1-42. Neuroscience.

[16] Mudò, G., M. Frinchi, D. Nuzzo, P. Scaduto, F. Plescia, M.F. Massenti, M. Di Carlo, C. Cannizzaro, G. Cassata, and L. Cicero (2019) Anti-inflammatory and cognitive effects of interferon-β1a (IFNβ1a) in a rat model of Alzheimer’s disease. Journal of Neuroinflammation 16(1). 10.1186/s12974-019-1417-4 10.1186/s12974-019-1417-4 PMC6380058 30777084

[17] He, W., K. Yuan, B. Ji, Y. Han, and J. Li (2020) Protective effects of curcumin against neuroin fl ammation induced by Abeta25-35 in primary rat microglia: modulation of high-mobility group box 1, toll-like receptor 4 and receptor for advanced glycation end products expression. Ann Transl Med 8(4): p. 88. 10.21037/atm.2019.12.147 10.21037/atm.2019.12.147 PMC7048970 32175381

[18] Shi CJ, Peng W, Zhao JH, Yang HL, Qu LL, Wang C, Kong LY, Wang XB (2020). Usnic acid derivatives as tau-aggregation and neuroinflammation inhibitors. Eur J Med Chem.

[19] Yan L, Wei M, Gong AG, Song P, Lou J, Bi CW, Xu SL, Xiong A, Dong TT, Tsim KW (2017). A modified Chinese herbal decoction (Kai-Xin-San) promotes NGF-induced neuronal differentiation in PC12 cells via up-regulating Trk A signaling. Front Cell Dev Biol.

[20] Xu YM, Wang XC, Xu TT, Li HY, Hei SY, Luo NC, Wang H, Zhao W, Fang SH, Chen YB, Guan L, Fang YQ, Zhang SJ, Wang Q, Liang WX (2019). Kai Xin San ameliorates scopolamine-induced cognitive dysfunction. Neural Regen Res.

[21] Fu H, Xu Z, Zhang XL, Zheng GQ (2019). Kaixinsan, a well-known Chinese herbal prescription, for Alzheimer’s disease and depression: a preclinical systematic review. Front Neurosci.

[22] Hu Y, Dong X, Zhang T, Ma H, Yang W, Wang Y, Liu P, Chen Y (2020). Kai-Xin-San suppresses matrix metalloproteinases and myocardial apoptosis in rats with myocardial infarction and depression. Mol Med Rep.

[23] Cao Y, Hu Y, Liu P, Zhao HX, Zhou XJ, Wei YM (2012). Effects of a Chinese traditional formula Kai Xin San (KXS) on chronic fatigue syndrome mice induced by forced wheel running. J Ethnopharmacol.

[24] Zhu Y, Duan X, Cheng X, Cheng X, Li X, Zhang L, Liu P, Su S, Duan JA, Dong TT, Tsim KW, Huang F (2016). Kai-Xin-San, a standardized traditional Chinese medicine formula, up-regulates the expressions of synaptic proteins on hippocampus of chronic mild stress induced depressive rats and primary cultured rat hippocampal neuron. J Ethnopharmacol.

[25] Lu C, Shi Z, Sun X, Pan R, Chen S, Li Y, Qu L, Sun L, Dang H, Bu L, Chen L, Liu X (2017). Kai Xin San aqueous extract improves Abeta1-40-induced cognitive deficits on adaptive behavior learning by enhancing memory-related molecules expression in the hippocampus. J Ethnopharmacol.

[26] Gao HL, Zhang AH, Yu JB, Sun H, Kong L, Wang XQ, Yan GL, Liu L, Wang XJ (2018). High-throughput lipidomics characterize key lipid molecules as potential therapeutic targets of Kaixinsan protects against Alzheimer’s disease in APP/PS1 transgenic mice. J Chromatogr B Analyt Technol Biomed Life Sci.

[27] Wang N, Jia Y, Zhang B, Li Y, Murtaza G, Huang S, Liu X (2020). Kai-Xin-San, a Chinese herbal decoction, accelerates the degradation of β-amyloid by enhancing the expression of neprilysin in rats. Evid Based Complement Alternat Med.

[28] Zhang AH, Ma ZM, Kong L, Gao HL, Sun H, Wang XQ, Yu JB, Han Y, Yan GL, Wang XJ (2020). High-throughput lipidomics analysis to discover lipid biomarkers and profiles as potential targets for evaluating efficacy of Kai-Xin-San against APP/PS1 transgenic mice based on UPLC-Q/TOF-MS. Biomed Chromatogr.

[29] Takeda T, Hosokawa M, Takeshita S, Irino M, Higuchi K, Matsushita T, Tomita Y, Yasuhira K, Hamamoto H, Shimizu K, Ishii M, Yamamuro T (1981). A new murine model of accelerated senescence. Mech Ageing Dev.

[30] Morley JE, Farr SA, Kumar VB, Armbrecht HJ (2012). The SAMP8 mouse: a model to develop therapeutic interventions for Alzheimer’s disease. Curr Pharm Des.

[31] Canudas AM, Gutierrez-Cuesta J, Rodríguez MI, D. Acua-Castroviejo, F.X. Sureda, A. Camins, and M. Pallàs,  (2005). Hyperphosphorylation of microtubule-associated protein tau in senescence-accelerated mouse (SAM). Mech Ageing Dev.

[32] Miyamoto M (1997). Characteristics of age-related behavioral changes in senescence-accelerated mouse SAMP8 and SAMP10. Exp Gerontol.

[33] Pallas M, Camins A, Smith MA, Perry G, Lee HG, Casadesus G (2008). From aging to Alzheimer’s disease: unveiling “the switch” with the senescence-accelerated mouse model (SAMP8). J Alzheimers Dis.

[34] Pallas M, Camins A, Smith MA, Perry G, Lee H-G, Casadesus G (2008). From aging to Alzheimer’s disease: unveiling “The Switch” with the senescence-accelerated mouse model (SAMP8). Journal of Alzheimer’s Disease.

[35] Ma Q, Qiang J, Gu P, Wang Y, Geng Y, Wang M (2011). Age-related autophagy alterations in the brain of senescence accelerated mouse prone 8 (SAMP8) mice. Exp Gerontol.

[36] Xu HY, Zhang YQ, Liu ZM, Chen T, Lv CY, Tang SH, Zhang XB, Zhang W, Li ZY, Zhou RR, Yang HJ, Wang XJ, Huang LQ (2019). ETCM: an encyclopaedia of traditional Chinese medicine. Nucleic Acids Res.

[37] Chu H, Zhang A, Han Y, Lu S, Kong L, Han J, Liu Z, Sun H, Wang X (2016). Metabolomics approach to explore the effects of Kai-Xin-San on Alzheimer’s disease using UPLC/ESI-Q-TOF mass spectrometry. Journal of chromatography. B, Analytical technologies in the biomedical and life sciences.

[38] Fei X (2017). Effects of Kaixin San on learning and memory ability of Alzheimer’s disease in rats. Clinical Journal of Chinese Medicine.

[39] Administration, F.D. (2005) Guidance for industry: estimating the maximum safe starting dose in initial clinical trials for therapeutics in adult healthy volunteers.

[40] Wang Z, Zhang YH, Zhang W, Gao HL, Zhong ML, Huang TT, Guo RF, Liu NN, Li DD, Li Y, Wang ZY, Zhao P (2018). Copper chelators promote nonamyloidogenic processing of AbetaPP via MT1/2 /CREB-dependent signaling pathways in AbetaPP/PS1 transgenic mice. J Pineal Res.

[41] Lovestone S, Reynolds CH, Latimer D, Davis DR, Anderton BH, Gallo JM, Hanger D, Mulot S, Marquardt B, Stabel S (1994). Alzheimer’s disease-like phosphorylation of the microtubule-associated protein tau by glycogen synthase kinase-3 in transfected mammalian cells. Curr Biol.

[42] Drummond E, Pires G, MacMurray C, Askenazi M, Nayak S, Bourdon M, Safar J, Ueberheide B, Wisniewski T (2020). Phosphorylated tau interactome in the human Alzheimer’s disease brain. Brain.

[43] He Y, Hara H, Nunez G (2016). Mechanism and regulation of NLRP3 inflammasome activation. Trends Biochem Sci.

[44] Guo S, Wang J, Xu H, Rong W, Gao C, Yuan Z, Xie F, Bi K, Zhang Z, Li Q (2019). Classic prescription, Kai-Xin-San, ameliorates Alzheimer’s disease as an effective multitarget treatment: from neurotransmitter to protein signaling pathway. Oxid Med Cell Longev.

[45] Wang N, Jia Y, Zhang B, Li Y, Murtaza G, Huang S, Liu X (2020). Kai-Xin-San, a Chinese herbal decoction, accelerates the degradation of beta-amyloid by enhancing the expression of neprilysin in rats. Evid Based Complement Alternat Med.

[46] Qiong W, Yong-Liang Z, Ying-Hui L, Shan-Guang C, Jiang-Hui G, Yi-Xi C, Ning J, Xin-Min L (2016). The memory enhancement effect of Kai Xin San on cognitive deficit induced by simulated weightlessness in rats. J Ethnopharmacol.

[47] Ishiguro K, Shiratsuchi A, Sato S, Omori A, Arioka M, Kobayashi S, Uchida T, Imahori K (1993). Glycogen synthase kinase 3 beta is identical to tau protein kinase I generating several epitopes of paired helical filaments. FEBS Lett.

[48] Yu Y, Run X, Liang Z, Li Y, Liu F, Liu Y, Iqbal K, Grundke-Iqbal I, Gong CX (2009). Developmental regulation of tau phosphorylation, tau kinases, and tau phosphatases. J Neurochem.

[49] Kopeikina KJ, Carlson GA, Pitstick R, Ludvigson AE, Peters A, Luebke JI, Koffie RM, Frosch MP, Hyman BT, Spires-Jones TL (2011). Tau accumulation causes mitochondrial distribution deficits in neurons in a mouse model of tauopathy and in human Alzheimer’s disease brain. Am J Pathol.

[50] Faraco G, Hochrainer K, Segarra SG, Schaeffer S, Santisteban MM, Menon A, Jiang H, Holtzman DM, Anrather J, Iadecola C (2019). Dietary salt promotes cognitive impairment through tau phosphorylation. Nature.

[51] Heng D, Wang Q, Ma X, Tian Y, Xu K, Weng X, Hu X, Liu W, Zhang C (2020). Role of OCT4 in the regulation of FSH-induced granulosa cells growth in female mice. Front Endocrinol.

[52] Shimizu T, Kagawa T, Inoue T, Nonaka A, Takada S, Aburatani H, Taga T (2008). Stabilized beta-catenin functions through TCF/LEF proteins and the Notch/RBP-Jkappa complex to promote proliferation and suppress differentiation of neural precursor cells. Mol Cell Biol.

[53] Su C, Yang C, Gong M, Ke Y, Yuan P, Wang X, Li M, Zheng X, Feng W (2019). Antidiabetic activity and potential mechanism of amentoflavone in diabetic mice. Molecules (Basel, Switzerland).

[54] Yamaguchi H, Ishiguro K, Uchida T, Takashima A, Lemere CA, Imahori K (1996). Preferential labeling of Alzheimer neurofibrillary tangles with antisera for tau protein kinase (TPK) I/glycogen synthase kinase-3 beta and cyclin-dependent kinase 5, a component of TPK II. Acta Neuropathol.

[55] Noble W, Olm V, Takata K, Casey E, Mary O, Meyerson J, Gaynor K, LaFrancois J, Wang L, Kondo T, Davies P, Burns M, Veeranna R, Nixon D, Dickson Y, Matsuoka M, Ahlijanian LFL, Duff K (2003). Cdk5 is a key factor in tau aggregation and tangle formation in vivo. Neuron.

[56] Czapski GA, Gassowska M, Songin M, Radecka UD, Strosznajder JB (2011). Alterations of cyclin dependent kinase 5 expression and phosphorylation in amyloid precursor protein (APP)-transfected PC12 cells. FEBS Lett.

[57] Tuo QZ, Liuyang ZY, Lei P, Yan X, Shentu YP, Liang JW, Zhou H, Pei L, Xiong Y, Hou TY, Zhou XW, Wang Q, Wang JZ, Wang XC, Liu R (2018). Zinc induces CDK5 activation and neuronal death through CDK5-Tyr15 phosphorylation in ischemic stroke. Cell Death Dis.

[58] Cheung ZH, Ip NY (2012). Cdk5: a multifaceted kinase in neurodegenerative diseases. Trends Cell Biol.

[59] Yan D, Yao J, Liu Y, Zhang X, Wang Y, Chen X, Liu L, Shi N, Yan H (2018). Tau hyperphosphorylation and P-CREB reduction are involved in acrylamide-induced spatial memory impairment: Suppression by curcumin. Brain Behav Immun.

[60] Guzman-Martinez L, Maccioni RB, Andrade V, Navarrete LP, Pastor MG, Ramos-Escobar N (2019). Neuroinflammation as a common feature of neurodegenerative disorders. Front Pharmacol.

[61] Morales, I., L. Guzmán-Martínez, C. Cerda-Troncoso, G.A. Farías, and R.B. Maccioni (2014) Neuroinflammation in the pathogenesis of Alzheimer’s disease. A rational framework for the search of novel therapeutic approaches. Frontiers in Cellular Neuroscience 8: p. 112. 10.3389/fncel.2014.00112 10.3389/fncel.2014.00112 PMC4001039 24795567

[62] Yang J, Wise L, Fukuchi KI (2020). TLR4 cross-talk with NLRP3 inflammasome and complement signaling pathways in Alzheimer’s disease. Front Immunol.

[63] Tahara K, Kim HD, Jin JJ, Maxwell JA, Li L, Fukuchi K (2006). Role of toll-like receptor signalling in Abeta uptake and clearance. Brain.

[64] Balducci C, Frasca A, Zotti M, La Vitola P, Mhillaj E, Grigoli E, Iacobellis M, Grandi F, Messa M, Colombo L, Molteni M, Trabace L, Rossetti C, Salmona M, Forloni G (2017). Toll-like receptor 4-dependent glial cell activation mediates the impairment in memory establishment induced by beta-amyloid oligomers in an acute mouse model of Alzheimer’s disease. Brain Behav Immun.

[65] Yang L, Zhou R, Tong Y, Chen P, Shen Y, Miao S, Liu X (2020). Neuroprotection by dihydrotestosterone in LPS-induced neuroinflammation. Neurobiol Dis.

[66] Rangasamy SB, Jana M, Roy A, Corbett GT, Kundu M, Chandra S, Mondal S, Dasarathi S, Mufson EJ, Mishra RK, Luan CH, Bennett DA, Pahan K (2018). Selective disruption of TLR2-MyD88 interaction inhibits inflammation and attenuates Alzheimer’s pathology. J Clin Invest.

[67] Bauernfeind FG, Horvath G, Stutz A, Alnemri ES, MacDonald K, Speert D, Fernandes-Alnemri T, Wu J, Monks BG, Fitzgerald KA, Hornung V, Latz E (2009). Cutting edge: NF-kappaB activating pattern recognition and cytokine receptors license NLRP3 inflammasome activation by regulating NLRP3 expression. J Immunol.

[68] Liang Z, Zhang B, Xu M, Morisseau C, Hwang SH, Hammock BD, Li QX (2019). 1-Trifluoromethoxyphenyl-3-(1-propionylpiperidin-4-yl) urea, a selective and potent dual inhibitor of soluble epoxide hydrolase and p38 kinase intervenes in Alzheimer’s signaling in human nerve cells. ACS Chem Neurosci.

[69] Miron J, Picard C, Lafaille-Magnan ME, Savard M, Labonte A, Breitner J, Rosa-Neto P, Auld D, Poirier J (2019). Association of TLR4 with Alzheimer’s disease risk and presymptomatic biomarkers of inflammation. Alzheimers Dement.

[70] Ising C, Venegas C, Zhang S, Scheiblich H, Schmidt SV, Vieira-Saecker A, Schwartz S, Albasset S, McManus RM, Tejera D, Griep A, Santarelli F, Brosseron F, Opitz S, Stunden J, Merten M, Kayed R, Golenbock DT, Blum D, Latz E, Buee L, Heneka MT (2019). NLRP3 inflammasome activation drives tau pathology. Nature.

[71] Stancu I-C, Cremers N, Vanrusselt H, Couturier J, Vanoosthuyse A, Kessels S, Lodder C, Brône B, Huaux F, Octave J-N, Terwel D, Dewachter I (2019). Aggregated Tau activates NLRP3–ASC inflammasome exacerbating exogenously seeded and non-exogenously seeded Tau pathology in vivo. Acta Neuropathol.

[72] Quintanilla RA (2004). Interleukin-6 induces Alzheimer-type phosphorylation of tau protein by deregulating the cdk5/p35 pathway. Exp Cell Res.

[73] Chakrabarty P, Jansen-West K, Beccard A, Ceballos-Diaz C, Levites Y, Verbeeck C, Zubair AC, Dickson D, Golde TE, Das P (2010). Massive gliosis induced by interleukin-6 suppresses Abeta deposition in vivo: evidence against inflammation as a driving force for amyloid deposition. FASEB J.

[74] Wang WY, Tan MS, Yu JT, Tan L (2015). Role of pro-inflammatory cytokines released from microglia in Alzheimer’s disease. Ann Transl Med.

[75] Luo Y, Li D, Liao Y, Cai C, Wu Q, Ke H, Liu X, Li H, Hong H, Xu Y, Wang Q, Fang J, Fang S (2020). Systems pharmacology approach to investigate the mechanism of Kai-Xin-San in Alzheimer’s disease. Front Pharmacol.

[76] Gu X, Cai Z, Cai M, Liu K, Liu D, Zhang Q, Tan J, Ma Q (2016). Protective effect of paeoniflorin on inflammation and apoptosis in the cerebral cortex of a transgenic mouse model of Alzheimer’s disease. Mol Med Rep.

[77] Mohammadzadeh L, Abnous K, Razavi BM, Hosseinzadeh H (2020). Crocin-protected malathion-induced spatial memory deficits by inhibiting TAU protein hyperphosphorylation and antiapoptotic effects. Nutr Neurosci.

[78] Obulesu M, Lakshmi MJ (2014). Apoptosis in Alzheimer’s disease: an understanding of the physiology, pathology and therapeutic avenues. Neurochem Res.

[79] Xie Q, Zhang S, Chen C, Li J, Wei X, Xu X, Xuan F, Chen N, Pham T, Qin N, He J, Ye F, Huang W, Huang R, Wen Q (2016). Protective effect of 2-dodecyl-6-methoxycyclohexa-2, 5-diene-1, 4-dione, isolated from Averrhoa carambola L., against palmitic acid-induced inflammation and apoptosis in Min6 cells by inhibiting the TLR4-MyD88-NF-kappaB signaling pathway. Cell Physiol Biochem.

[80] Wei X, Xu X, Chen Z, Liang T, Wen Q, Qin N, Huang W, Huang X, Li Y, Li J, He J, Wei J, Huang R (2018). Protective effects of 2-dodecyl-6-methoxycyclohexa-2,5 -diene-1,4-dione isolated from Averrhoa carambola L. (Oxalidaceae) roots on neuron apoptosis and memory deficits in Alzheimer’s disease. Cell Physiol Biochem.

[81] Wang LY, Yu X, Li XX, Zhao YN, Wang CY, Wang ZY, He ZY (2019). Catalpol exerts a neuroprotective effect in the MPTP mouse model of Parkinson’s disease. Front Aging Neurosci.

[82] Shen X, Venero JL, Joseph B, Burguillos MA (2018). Caspases orchestrate microglia instrumental functions. Prog Neurobiol.

[83] Wang CY, Xu Y, Wang X, Guo C, Wang T, Wang ZY (2019). Dl-3-n-Butylphthalide inhibits NLRP3 inflammasome and mitigates Alzheimer’s-like pathology via Nrf2-TXNIP-TrX axis. Antioxid Redox Signal.

[84] Saadi M, Karkhah A, Pourabdolhossein F, Ataie A, Monif M, Nouri HR (2020). Involvement of NLRC4 inflammasome through caspase-1 and IL-1beta augments neuroinflammation and contributes to memory impairment in an experimental model of Alzheimer’s like disease. Brain Res Bull.

[85] Serpente M, Bonsi R, Scarpini E, Galimberti D (2014). Innate immune system and inflammation in Alzheimer’s disease: from pathogenesis to treatment. NeuroImmunoModulation.

