# Supplementary material for: Kai-Xin-San Inhibits Tau Pathology and Neuronal Apoptosis in Aged SAMP8 Mice
Source: Mol Neurobiol. 2022 Mar 18;59(5):3294–309. doi: 10.1007/s12035-021-02626-0 (PMC9016055; doi:10.1007/s12035-021-02626-0)
Supplement: Supplementary file 1 — Supplementary file1 (DOCX 2743 KB) [file 12035_2021_2626_MOESM1_ESM.docx]

Kai-Xin-San Inhibits Tau Pathology and Neuronal Apoptosis in Aged SAMP8 Mice

Ya-Nan Jiao^a^, Jing-Sheng Zhang^b^, Wen-Jun Qiao^b^, Shu-Yu Tian^a^, Yi-Bin Wang^a^, Chun-Yan Wang^a^, Yan-Hui Zhang^c^, Qi Zhang^a^, Wen Li^a^, Dong-Yu Min^b,^*, Zhan-You Wang^a,^*

^a^ Institute of Health Sciences, China Medical University, Shenyang, China

^b^ Affiliated Hospital of Liaoning University of Traditional Chinese Medicine, Shenyang, China

^c^ School of Fundamental Sciences, China Medical University, Shenyang, China

* Corresponding author.

E-mail address: Wangzy@cmu.edu.cn (Z. Wang), yangyongju@lnutcm.edu.cn (D. Min)

a

b

Supplementary Fig. 1 HPLC characteristic chromatogram of Kai-Xin-San (KXS)

HPLC chromatogram of (a) reference substances and (b) KXS. HPLC peaks; the No.1 peak represents Ginsenoside Rg1 and the No.2 peak represents Ginsenoside Rb1. The HPLC characteristic chromatogram were analyzed using Agilent 1200 HPLC system (Agilent Technologies, Boeblingen, Germany). The KXS (5mL) was extracted 3 times with 20 mL of water-saturated n-butanol. The mixed n-butanol phase was evaporated to dryness, solubilized with methanol, diluted to 5 mL with methanol, and shaken well. Filtered and the filtrate was collected for further application. The chromatographic separation was performed using a Agilent TC-C18（4.6×250mm, 5μm）column at 30℃, and acetonitrile (A) and water (B) were used as the mobile phase for analysis. The flowrate was set at 1.0 mL/min. The elution conditions were applied with a gradient program as follows: 19 % A, 81 % B for 0-35 min; 19-29% A, 81-71 % B for 35-55 min; 29 % A, 71 % B for 55-70 min; 29-40 % A, 71-60 % B for 70-100 min. The HPLC injection volume was 5μL, and the detection wavelength was 203 nm.


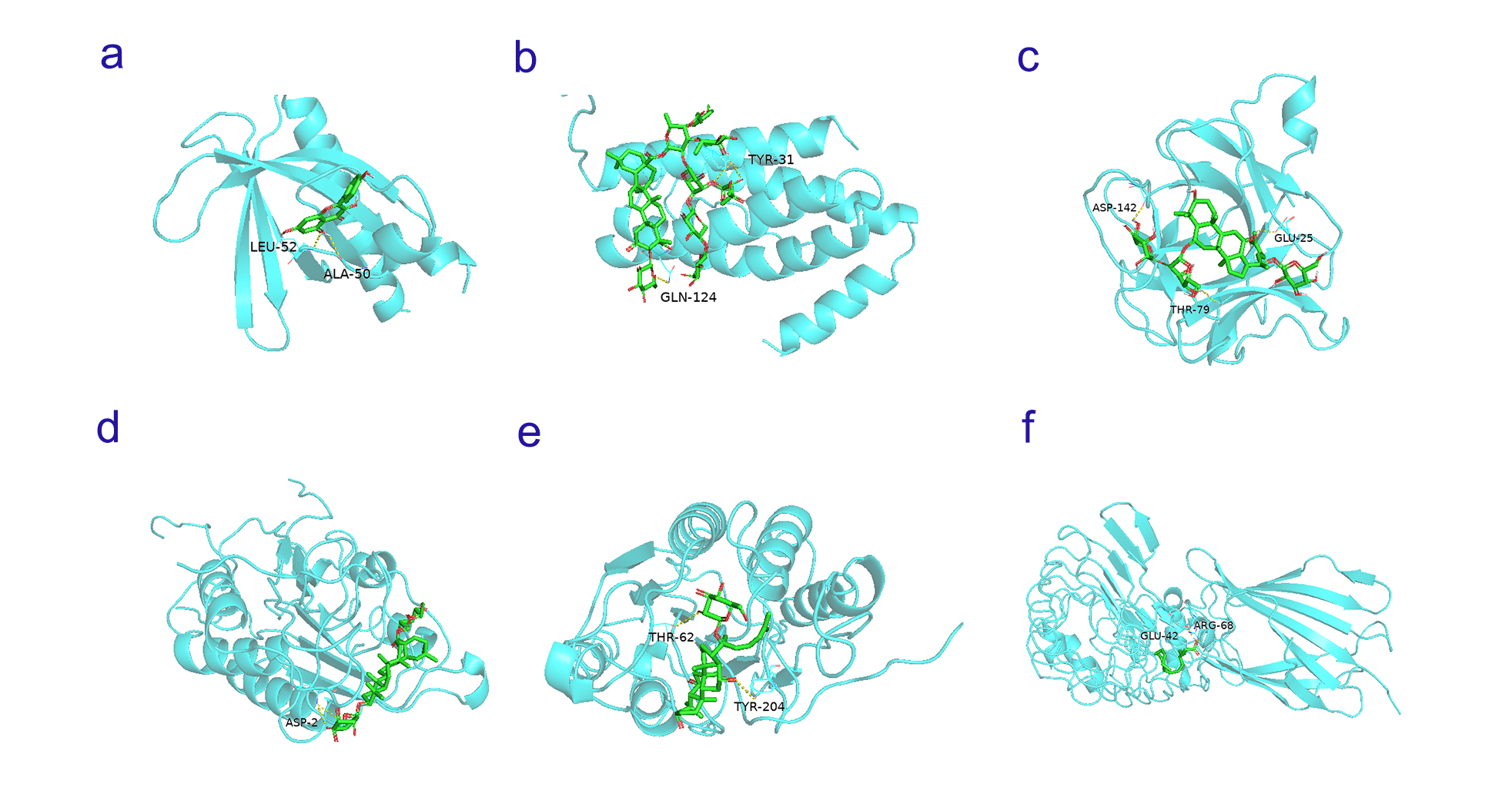


**Supplementary Fig. 2** The docking model of compounds with Tagerts. (a-f) Molecular models of the binding of Kaempferol, Onjisaponin A, Ginsenoside Ii, Chikusetsusapon, Ginsenoside F1, and Cis-9, Cis-12-Linoleic-Acid from KXS to the predicted targets AKT, IL-6, IL-1β, caspase 3 and TLR4 with the highest scores of -6.0, -7.4, -8.9, -9.5, -9.5 and -6.0 kcal/mol, respectively.


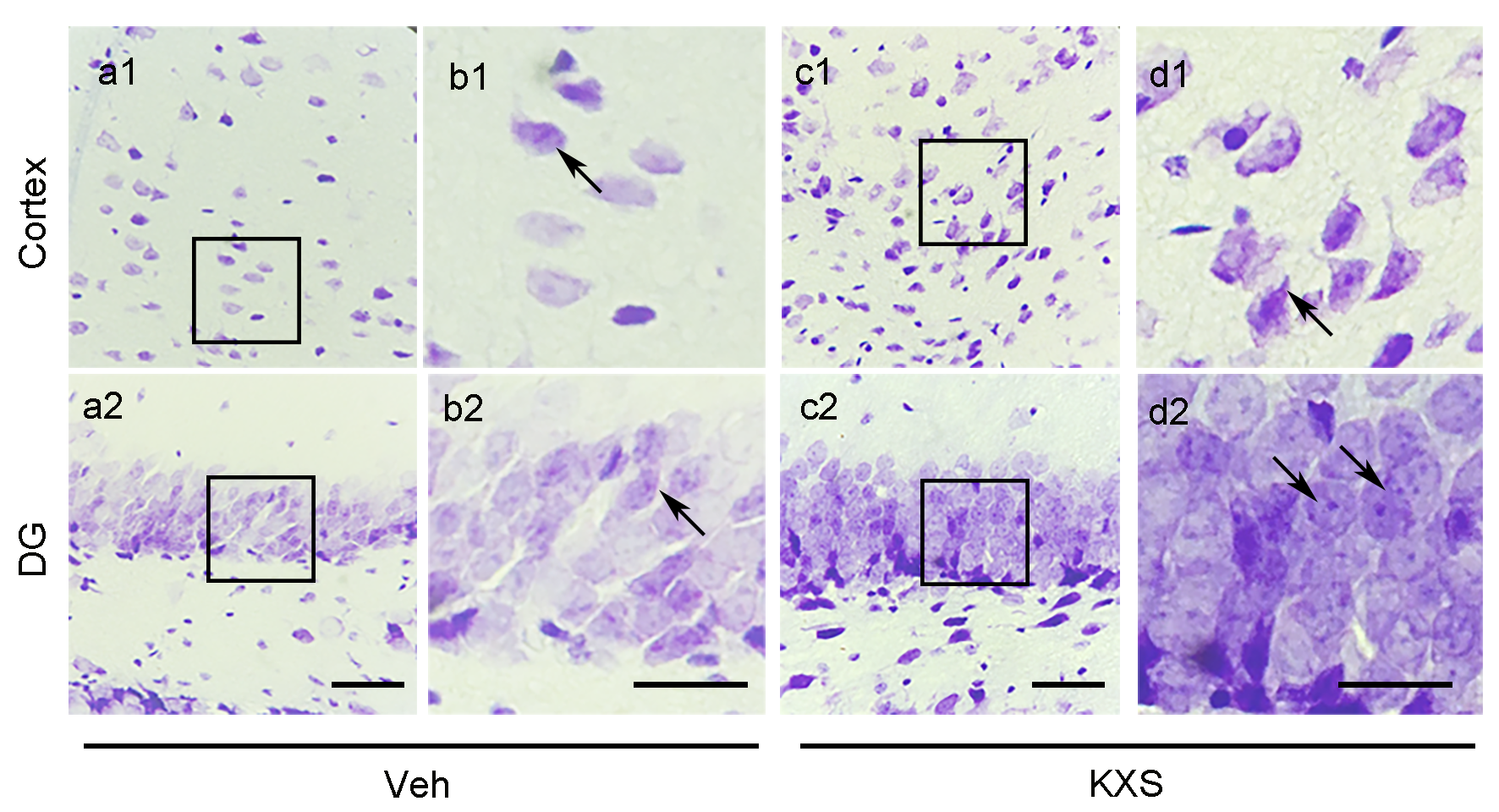


Supplementary Fig. 3 Cresyl violet stains showing the Nissl body in the neuronal cells of the cerebral cortex and hippocampal DG regions. Scale bars: 60 μm. Scale bar is 30 μm in the high magnification of right panels. The numbers of Nissl body indicate the surviving neurons.

Supplementary Fig. 4 Swimming speed in the MWM. Both groups had similar swimming speed.
